# Whole-Genome Sequencing and Potassium-Solubilizing Mechanism of *Bacillus aryabhattai* SK1-7

**DOI:** 10.3389/fmicb.2021.722379

**Published:** 2022-01-04

**Authors:** Yifan Chen, Hui Yang, Zizhu Shen, Jianren Ye

**Affiliations:** Co-Innovation Center for Sustainable Forestry in Southern China, College of Forestry, Nanjing Forestry University, Nanjing, China

**Keywords:** *Bacillus aryabhattai*, whole genome, mechanism of potassium solubilization, real-time fluorescence quantitative PCR, potassium-solubilizing bacteria (KSB)

## Abstract

To analyze the whole genome of *Bacillus aryabhattai* strain SK1-7 and explore its potassium solubilization characteristics and mechanism, thus providing a theoretical basis for analyzing the utilization and improvement of insoluble potassium resources in soil. Genome information for *Bacillus aryabhattai* SK1-7 was obtained by using Illumina NovaSeq second-generation sequencing and GridION Nanopore ONT third-generation sequencing technology. The contents of organic acids and polysaccharides in fermentation broth of *Bacillus aryabhattai* SK1-7 were determined by high-performance liquid chromatography and the anthrone sulfuric acid method, and the expression levels of the potassium solubilization-related genes *ackA*, *epsB*, *gltA*, mdh and *ppc* were compared by real-time fluorescence quantitative PCR under different potassium source culture conditions. The whole genome of the strain consisted of a complete chromosome sequence and four plasmid sequences. The sequence sizes of the chromosomes and plasmids P1, P2, P3 and P4 were 5,188,391 bp, 136,204 bp, 124,862 bp, 67,200 bp and 12,374 bp, respectively. The GC contents were 38.2, 34.4, 33.6, 32.8, and 33.7%. Strain SK1-7 mainly secreted malic, formic, acetic and citric acids under culture with an insoluble potassium source. The polysaccharide content produced with an insoluble potassium source was higher than that with a soluble potassium source. The expression levels of five potassium solubilization-related genes with the insoluble potassium source were higher than those with the soluble potassium source.

## Introduction

Potassium (K) is an essential element for plant nutrition and plays an important role in the growth and metabolism of plants. Additionally, potassium can improve cold, drought, and stress resistance and promote photosynthesis in plants (Leigh and Wyn Jones, 1984; Marschner, 1995; Schachtman and Shin, 2007; Wang M. et al., 2013). The potassium content (K_2_O) in crops is 0.3–5% (dry weight), which is equivalent to that of nitrogen and higher than that of phosphorus. Most of the potassium in soil exists in the form of insoluble potassium, such as potassium feldspar, mica and illite, which cannot be directly utilized by plants (Meena et al., 2014). At present, chemical potassium fertilizer is mainly used to increase the available potassium in soil, but long-term application of chemical potassium fertilizer to soil can lead to the imbalance of sodium and potassium in soil and cause destructive effects on a number of soil microorganisms, affect the pH value and weaken the soil structure (Abbiramy and Ross, 2013).

Potassium-solubilizing bacteria (KSB), also known as silicate bacteria, are bacteria isolated from soil that can decompose aluminosilicate and apatite minerals. These organisms can transform insoluble potassium, phosphorus, silicon and other substances in soil into soluble substances and have the advantages of offering environmental protection with low cost and high efficiency (Ahmad et al., 2016; Bagyalakshmi et al., 2017; Ji et al., 2017; Khanghahi et al., 2018; Bahadur et al., 2019). In addition to transforming insoluble potassium in soil into soluble potassium that can be easily absorbed by plants, KSB can also improve the physical and chemical properties of soil and secrete hormones that promote plant growth to increase plant biomass (Zhang and Kong, 2014).

According to the existing research, the potassium decomposition process of KSB (the decomposition of potassium-containing aluminosilicate minerals by such bacteria) is completed by various mechanisms, including acidolysis, chelation, dissolution, active absorption, etc., which promote each other and eventually lead to the gradual precipitation of K^+^ in minerals (Du et al., 2008; Zhou et al., 2010; Hu et al., 2011; Lian et al., 2020).

*Bacillus aryabhattai* exists widely in soil. Thus far, research on this kind of bacteria has mainly focused on two areas: degradation and synthetic application and plant protection. Existing studies have confirmed that *B. aryabhattai* can coexist with plants or even promote the growth of plants (Xiong et al., 2018), such as corn, rice, soybean and other plants (Park and Raddhakrishnan, 2015; Kavamura et al., 2017; Park et al., 2017). This species may protect plants from the toxic effects of arsenic, copper, lead and nitrite (Paredes-Páliz et al., 2016; Singh et al., 2016) and control plant diseases (Li et al., 2014; Jiang and Hu, 2016). Additionally, *B. aryabhattai* synthesizes the biological hormone IAA, dissolves phosphorus and fixes nitrogen to promote plant growth (Bhattacharyya et al., 2017; Zhou et al., 2017; Deng et al., 2018).

In our previous research, we selected the KSB *B. aryabhattai* SK1-7 from the rhizosphere of poplar trees (Xi and Ye, 2020). The results showed that the strain could dissolve insoluble potassium and release soluble potassium ions, and it could promote the growth of poplar after being applied to the rhizosphere soil, and the concentration of potassium dissolved reached 10.8 μg/mL and the percentage of potassium released was 32.6% (Chen et al., 2020). However, the mechanism of potassium solubilization of this strain is not clear. Therefore, in this study, to accurately locate potassium solubilization-related genes, the whole genome of this strain was sequenced by combining second- and third-generation sequencing methods, potassium solubilization-related genes were mined, the content of organic acids and polysaccharides produced by this strain under culture with an insoluble potassium source was determined, and the expression patterns of potassium solubilization-related genes under culture with different potassium sources were analyzed, thus revealing the potassium solubilization mechanism of the SK1-7 strain at the molecular level. These studies will undoubtedly contribute to a more comprehensive understanding of the potassium solubilization characteristics and growth-promoting ability of strain SK1-7 and provide a theoretical basis for further exploring the development and application of strain SK1-7 as a microbial fertilizer.

## Materials and Methods

### Strains

*Bacillus aryabhattai* SK1-7 was isolated from the rhizosphere of *Populus alba* L. and preserved at the Laboratory of Forest Pathology, Nanjing Forestry University.

### Culture Media for the SK1-7 Strain

Luria–Bertani (LB) medium was composed of 10.0 g of tryptone, 5.0 g of yeast extract, 10.0 g of NaCl, and 1,000 mL of deionized water (pH 7.2).

Two groups of fermentation media with different potassium sources were set up. Fermentation medium A had an insoluble potassium source (potassium feldspar) and was composed of the following: sucrose 10.0 g, Na_2_HPO_4_ 1 g, MgSO_4_⋅7H_2_O 1 g, FeCl_3_ 0.0005 g, (NH_4_)_2_SO_4_ 0.5 g, yeast 0.2 g, potassium feldspar powder 12 g, deionized water 1,000 mL, pH value 7.2 Fermentation medium group B was supplemented with a soluble potassium source (K_2_HPO_4_): the potassium feldspar powder in group A was replaced by 0.1% K_2_HPO_4_, and the other components remained unchanged.

Potassium feldspar was purchased from Rongshide Co., Ltd. (Hefei, China), ground and sieved, soaked in a hydrochloric acid solution for 24 h, washed with deionized water, and dried for later use.

### Genome Sequencing and Analysis of Strain SK1-7

#### Sample Preparation for Genome Sequencing of Strain SK1-7

The tested strains were inoculated into LB culture medium at 30°C and 200 r/min and cultured to the logarithmic growth stage with an OD600 value of 0.6. The culture medium was centrifuged at 10,000 r/min at 4°C for 10 min, and the supernatant was removed. Then, 1 × PBS buffer was applied as a wash 3–4 times until the supernatant was clear, and the samples were stored in a refrigerator at −80°C.

#### Genome Sequencing, Assembly, and Annotation

The samples were sent to a sequencing company (Personal, Shanghai), and the extracted and tested qualified total DNA samples of *B. aryabhattai* SK1-7 were sequenced. A5-miseq v20150522 and SPAdesv3.9.0 were used to assemble the sequencing data without linker sequences from scratch, the assembly effects were compared, and the results of SPAdes software analyses were selected for construction. GeneMarkerS (version 4.32 April 192015) software was used to predict the whole gene sequence. The TRNA gene was predicted by tRNAscan-SE (version 1.3.1), and the rRNA gene was predicted by Barrnap (0.9-dev). DRs (forward repeats) and spacers (spacers) in the whole genome were predicted by CRISPR finder.^1^ Sequence alignment of protein-coding genes was completed by blastall software.

### Determination of Organic Acids Produced by Strain SK1-7

The tested strains were inoculated into LB medium at 30°C and 200 r/min and cultured to the logarithmic growth stage as seed solution. The seed solution was inoculated into 20 mL fermentation medium with the insoluble potassium source (potassium feldspar) with a 5% inoculation amount and 3 replicates in each group. In addition, LB medium with the same volume was inoculated as a blank control and cultured at 30°C and 200 r/min for 168 h. Samples were taken at 24, 96, and 168 h, the fermentation broth was centrifuged at 8,000 r/min for 10 min, and 5 mL supernatant was collected. The types and contents of organic acids in the potassium-decomposing fermentation broth inoculated with strain SK1-7 were determined by high-performance liquid chromatography with three replicates. Chromatographic conditions were as follows: Agilent InfinityLab Poroshell120 SB-C8, 4.6 mm × 100 mm, 2.7 μm, mobile phase 0.02 mol/L NH_4_H_2_PO_4_-H_3_PO_4_ (pH value 2.9), flow rate 0.4 mL/min, column temperature 30°C, detection wavelength 210 nm. Organic acid standards were as follows: oxalic acid, citric acid, succinic acid, fumaric acid, tartaric acid, formic acid, acetic acid, gluconic acid and malic acid.

### Determination of the Polysaccharide Contents of Strain SK1-7 Cultured With Different Potassium Sources

The tested strains were inoculated into LB medium at 30°C and 200 r/min and cultured to the logarithmic growth stage as seed solution. The seed solution was inoculated with 5% inoculum into two groups, fermentation media A and B with different potassium sources, and each group was set up with three replicates. The groups were cultured with shaking at 30°C and 200 r/min. Samples were taken at 24, 48, 72, 96, 120, 144, and 168 h, the fermentation broth was centrifuged at 8,000 r/min for 10 min, and 5 mL supernatant was collected. Then, 15 mL absolute ethyl alcohol was added and precipitated at 4°C for 2 h, the mixture was centrifuged at 8,000 r/min for 10 min and the supernatant was removed. Then, 1.0 mL absolute ethyl alcohol was added to precipitate polysaccharides, and the mixture was centrifuged at 8,000 r/min for 10 min and the supernatant was removed. Next, 4.0 mL deionized water was added for precipitation, and the polysaccharide precipitate was dissolved. Then 1.0 mL Sevag solution was added to remove protein under continuous mixing for 30 min followed by standing until layering was observed. The sample was centrifuged for 10 min at 8,000 r/min, and the volume of the supernatant was adjusted to 10 mL, followed by the addition of 1 mL deionized water to 4.0 mL. Then, 6 mL sulfuric acid-anthrone solution was added, the mixture was placed in boiling water for 10 min and cooled with running water after treatment. A standard curve was established as follows. First, 200 mg of anhydrous glucose standard was mixed with deionized water to a constant volume of 1,000 mL and shaken well. Then, 0, 0.2, 0.4, 0.6, 0.8, and 1.0 mL of glucose standard solution were removed by suction, and deionized water was added to keep the volume constant at 4.0 mL. Then, 6.0 mL of sulfuric acid-anthrone solution was added, and the solution was shaken in boiling water for 10 min. After treatment, the solution was cooled with cold water, and its absorbance was measured at 625 mm wavelength (Ma, 2011).

### Expression Patterns of Potassium Solubilization-Related Genes in *Bacillus aryabhattai* SK1-7 Cultured With Insoluble Potassium Sources

#### Total RNA Extraction and cDNA Synthesis of Strain SK1-7

The tested strains were inoculated into LB medium at 30°C and 200 r/min and cultured to the logarithmic growth stage as seed solution. The seed solution was inoculated with 5% inoculum into two groups, fermentation media A and B with different potassium sources, and each group was set up with three replicates, which were cultured with shaking at 30°C and 200 r/min. When the culture time was 4, 6, 8, 10, and 12 h, the fermentation broth was centrifuged at 4°C for 10 min at 10,000 r/min, the supernatant was removed, 200 μL lysozyme was added, and the mixture was placed in a 37°C metal bath for 10 min. Then a total RNA Extraction Kit (Beijing Tianmo Technology Development Co., Ltd., Beijing) was used to extract the strain SK1-7 RNA; all equipment used in the extraction process such as centrifuge tubes, pipette tips, etc. were treated with 0.1% diethyl carbonate (DEPC) and sterilized. The total RNA mass of the extracted bacteria was detected by agarose gel electrophoresis at a concentration of 1%, and the total RNA concentration and purity were determined by a Nanodrop series ultramicro spectrophotometer (Thermo Fisher Scientific, Waltham, United States). Using an RNA reference reverse transcription kit (Accurate Biology Co., Ltd., Changsha), 1 μg of total RNA was taken to prepare the reaction solution and reacted in a PCR instrument (Eppendorf no. 5345/015458, Germany). The reverse transcribed cDNA concentration was diluted to approximately 100 ng/μL and stored at 4°C until use.

#### Real-Time Fluorescent Quantitative PCR of Potassium Solubilization-Related Genes Under Insoluble Potassium Source Culture Conditions

To understand the potassium solubilizing mechanism of strain SK1-7 from multiple perspectives, the relative expression levels of SK1-7 genes under different potassium sources were determined by real-time quantitative PCR. The control group was group B supplemented with a soluble potassium source (K_2_HPO_4_), and the experimental group was group A supplemented with an insoluble potassium source (potassium feldspar). Different potassium solubilizing genes were selected: genes *ackA, epsB, gltA, mdh*, and *ppc*. Primer Premier 5.0 software was used to design specific primers for real-time fluorescence quantitative PCR. The specific primer design is shown in Table 1. A SYBR green *Pro Tap* Hs premixed qPCR (Low ROX Premixed) kit (Accurate Biology Co., Ltd., Changsha) and 7900 real-time system qRT-PCR instrument were used to perform qRT-PCR, according to the instructions of the kit, and the reaction solution was prepared on ice by pressing the system. The gyrA gene was used as the internal reference gene, and sterile water was used instead of the template for expansion as the negative control. The amplification reaction adopted a two-step amplification procedure: Stage 1: predenaturation, reps: 1, 95°C, 30 s; Stage 2: cyclic reaction, reps: 40, 95°C, 5 s, 60°C, 30 s; Stage 3: melting curve, reps: 95°C, 15 s, 60°C, 15 s; 95°C, 15 s. In this RT-qPCR reaction, three samples were set up for each potassium source, and three technical repetitions were set up for each sample. The data obtained from the reaction were analyzed by one-way ANOVA, the gene expression was calculated and analyzed, and the relative gene expression under different potassium source culture conditions was calculated by the 2^–△^^△^^△^^ CT^ method (Livak and Schmittgen, 2001).

**TABLE 1:** Specific primers for qRT-PCR of *Bacillus aryabhattai* SK1-7 solubilization-related genes.

Gene name	Primer (5′-3′)
*ackA*	F:CAATGAACGCGCTGAAACAG
	R:GAACACGAGCACGTACAACA
*epsB*	F:TGCTGTTTATGCCCAGCAAG
	R:ATAATGTGCGGTCGGCTTTC
*gltA*	F:CTTACACGCTGACCATGAGC
	R:AGGACCCTTTAACGCTCCAA
*mdh*	F:TGACAAACCCGGTAGATGCT
	R:CGTACGGAAACGTGCAGAAT
*ppc*	F:CGCGAATATCAGCAGCAAGA
	R:GGCTTCTGTTGGATGAGCAG
*gyrA*	F:GATATGCGCCTACAGCGTTT
	R:GCTCCTCCGCTTACGATTTC

### Statistical Analyses

One-way analysis of variance (ANOVA, Duncan) was performed using SPSS; different letters indicate significant differences (*p* < 0.05).

## Results

### Genome Assembly and Annotation of Strain SK1-7

**FIGURE 1:**
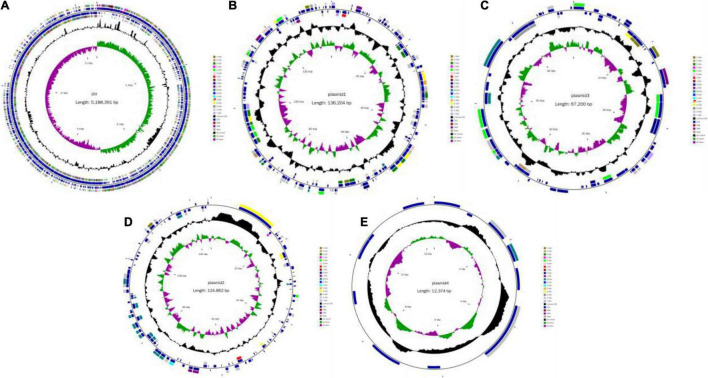
Circular genome map of *Bacillus aryabhattai* SK1-7. **(A)** Genetic map of the circular chromosome. **(B)** Cycle map of Plasmid1. **(C)** Cycle map of Plasmid 2. **(D)** Cycle map of Plasmid 3. **(E)** Cycle map of Plasmid 4. The circles for chromosome from the outside to the center represent CDS, COG, KEGG, GO, tRNA, rRNA, GC content, and GC skew, respectively.

The original reading of the SK1-7 strain obtained by sequencing was used for quality control, quality evaluation and assembly. The whole genome of the strain consisted of a circular chromosome and four circular plasmid (Figure 1). The length of the chromosome was 5,188,391 bp, and the GC content was 38.2%. The genome encoded 5,307 genes, accounting for 81% of the genome. The total length of the coding genes was 4,207,257 bp, the average length of the coding genes was 792.78 bp, the size of N50 was 18,721 bp, and the size of N90 was 2,442 bp, a total of 5,307 ORFs were predicted in the chromosome, and the length of the ORFs was 4,207,257 (Table 2). The genome of strain SK1-7 predicted 120 tRNA structures, 323 ncRNA structures, 14 5S rRNA structures, 13 16S rRNA structures, 13 23S rRNA structures and 4 CRISPR structures (Table 2). Strain SK1-7 was found to four plasmids, P1, P2, P3 and P4, with sequence sizes of 136,204 bp, 124,862 bp, 67,200 bp and 12,374 bp, and the GC contents were 34.4, 33.6, 32.8, and 33.7%, a total of 348 ORFs were predicted in all plasmid of which 313 were putative protein-coding DNA sequences (CDS), respectively (Table 3). Genome sequencing data of *B. aryabhattai* SK1-7 were submitted to NCBI with the GenBank BioProject number: PRJNA716807.

**TABLE 2:** Statistics of the genomic characteristics of *B. aryabhattai* SK1-7.

Name	Numerical value	Name	Numerical value
Genomic size (bp)	5,188,391	N50 (bp)	18,721
GC content (%)	38.2	N90 (bp)	2,442
ORF number	5307	ORF total length (bp)	4,207,257
tRNA	120	ncRNA	323
CRISPR	4	5S rRNA	14
16S rRNA	13	23S rRNA	13

**TABLE 3:** Statistics of the genomic characteristics of *B. aryabhattai* SK1-7.

Name	Seq length (bp)	GC content (%)	ORF number	CDS
Plasmid 1	136,204	34.4	154	140
Plasmid 2	124,862	33.6	113	104
Plasmid 3	67,200	32.8	69	57
Plasmid 4	12,374	33.7	12	12

### Functional Annotation of the Protein-Coding Genes of Strain SK1-7

The main purpose of functional annotation of protein-coding genes is to analyze the function of all protein-coding genes to deeply examine a species at the molecular level. According to the functional annotation results of the protein-coding genes of the strain SK1-7 genome (Table 4), 5,159 protein-coding genes were compared in the NR database, and 2,513 protein-coding genes were compared in the KEGG database. The differences were mainly related to the capacity and focus of the databases.

**TABLE 4:** Functions of the protein-coding genes of *B. aryabhattai* SK1-7.

Annotation database	No. of genes	Percentage of total (%)
NR	5,159	97.2112
eggNOG	4,427	83.4181
KEGG	2,513	47.3526
Swiss-Prot	4,004	75.4475
GO	3,596	67.7596

### Determination of Organic Acid Production by Strain SK1-7

As shown in Table 5, the organic acid production of strain SK1-7 was the highest under the insoluble potassium source culture, with 183.14 ng/μL at 168 h, followed by formic acid, citric acid, acetic acid and gluconate, with yields at 168 h of 81.68, 40.27, 38.63 and 36.39 ng/μL; small amount of fumaric acid and oxalic acid were also detected. Thus, it can be suggested that under the condition of an insoluble potassium source, the strain could secrete many organic acids, which were presumed to play a role in the process of potassium solubilization.

**TABLE 5:** The organic acid contents in fermentation broth after *B. aryabhattai* SK1-7 inoculation.

Organic acid (ng/μ L)	CK	SK1-7		
		24 h	96 h	168 h
Oxalic acid	−	16.74 ± 1.01	−	−
Citric acid	−	42.29 ± 3.24	37 ± 2.36	40.27 ± 3.47
Butanedioic acid	127.71 ± 1.4	−	−	−
Fumaric acid	−	0.4 ± 0.01	0.11 ± 0.01	0.24 ± 0.02
Tartaric acid	0.4 ± 0.03	−	−	−
Formic acid	8.38 ± 0.6	63.9 ± 1.6	84.2 ± 2.36	81.68 ± 3.44
Acetic acid	6.62 ± 0.12	40.79 ± 1.32	44.56 ± 2.1	38.63 ± 1.07
Gluconic acid	−	86.03 ± 2.17	45.62 ± 1.67	36.39 ± 1.98
Malic acid	0.854 ± 0.01	188.69 ± 6.23	189.68 ± 8.79	183.14 ± 7.35

### Determination of Polysaccharide Yields of Strain SK1-7 Cultured With Different Potassium Sources

As shown in Table 6, the yield of polysaccharides was higher than that of soluble potassium under the condition of an insoluble potassium source. The polysaccharide yield of strain SK1-7 increased gradually after 24–144 h of culture with an insoluble potassium source and then decreased to 5.1 mg/mL^–1^ after reaching the highest value of 6.82 mg/mL^–1^ 168 h at 144 h. Strain SK1-7 can produce a large amount of polysaccharides under culture with an insoluble potassium source. This may be because during the process of potassium solubility, bacteria secrete polysaccharides and feldspar to form a bacteria-mineral complex and then secrete acidic substances for acid dissolution.

**TABLE 6:** Changes in the polysaccharide contents of *B. aryabhattai* SK1-7 under different potassium sources.

Incubation time (h)	Polysaccharide content (mg/mL^–1^)	
	Soluble potassium source (K_2_HPO_4_)	Insoluble potassium source (potassium feldspar)
24	1.31	2.12
48	2.93	3.65
72	3.41	4.98
96	3.94	5.44
120	4.55	6.34
144	5.16	6.82
168	4.43	5.1

### Expression Analysis of Potassium Solubilizing Genes in Strain SK1-7

**FIGURE 2:**
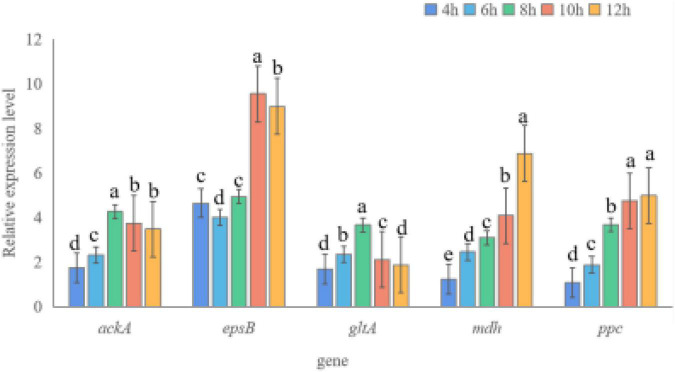
Expression levels of potassium solubilization genes in *B. aryabhattai* SK1-7. Different letters above the bars indicate significant differences (*p* < 0.05). The error bars indicate standard errors (*n* = 3).

To understand the role of potassium solubilization-related genes of strain SK1-7 from multiple perspectives, potassium solubilization-related genes were selected and tested by qRT-PCR under different potassium sources. As shown in Figure 2, the expression levels of the five genes in KSB in the fermentation medium supplemented with an insoluble potassium source (potassium feldspar) were higher than those of KSB in the fermentation medium supplemented with a soluble potassium source (K_2_HPO_4_). In the first 8 h, the expression of the *ackA* gene increased with increasing culture time; the expression was 4.26 times at 8 h and decreased after 10 h, while it was 3.48 times at 12 h. The expression of *epsB* increased from 4 to 10 h, and was 9.55 times and 8.99 times at 10 h and 12 h, respectively. The expression of *gltA* increased from 1.67 times to 3.66 times at 4–8 h and decreased to 1.06 times and 1.02 times at 10–12 h. The expression of *mdh* increased gradually from 1.23 times to 6.88 times at 4–12 h. The *ppc gene* also increased with increasing culture time, and the highest expression level was 4.99 times at 12 h.

## Discussion

Potassium bacteria, also known as potassium-solubilizing bacteria and silicate bacteria, can decompose aluminosilicate rocks in soil, and the decomposed nutrients can be used for plant growth (Saha et al., 2016). At present, many researchers have paid attention to the screening of high-efficiency potassium solubilizing strains, but there is still a lack of potassium bacteria strains with stable and high-efficiency potassium solubilizing effects and clear potassium solubilizing mechanisms, which seriously restricts the development of these potassium bacteria. In the research and application of plant rhizosphere growth-promoting bacterial mechanisms, with the help of genome sequencing technology, we can carry out in-depth analyses of gene regulation mechanisms and expression (Liu et al., 2020).

In this study, a complete chromosomal sequence and four plasmid sequences of the whole genome of *B. aryabhattai* SK1-7 were determined. The length of the chromosome was 5,188,391 bp, the GC content was 38.2%, the N50 was 18,721 bp, and the N90 was 2,442 bp. The chromosome encoded 5,307 genes, accounting for 81% of the genome length. The total length of the coding genes was 4,207,257 bp. Yan et al. (2016) sequenced the whole genome of *B. aryabhattai* T61, the total length of the genome was 5,325,933 bp, and the GC content was 38%. A total of 5,534 genes were encoded, accounting for 83.4% of the genome length. Bhattacharyya et al. (2017) sequenced the whole genome of *B. aryabhattai* AB211, the circular chromosome was 5,403,026 bp, and the GC content was 37.8%, a total of 5,468 ORFs were predicted in the genome of which 5,226 were putative protein-coding DNA sequences (CDS). The sequencing results of these strain were similar to those of the SK1-7 genome in this study.

In a previous study, strain SK1-7 was inoculated into poplar rhizosphere soil. The results showed that the pH value of rhizosphere soil inoculated with SK1-7 was lower than that of the blank control. These results indicate that SK1-7 may secrete acidic substances during the growth process, reduce the pH value of rhizosphere soil, and convert the insoluble potassium in soil into soluble potassium that can be absorbed and utilized by plants, thus increasing the content of available potassium in the rhizosphere soil and promoting the growth of poplar (Chen et al., 2020). In this study, strain SK1-7 mainly produces malic acid, formic acid, citric acid, acetic acid and gluconic acid under culture with an insoluble potassium source. Most scholars believe that the production of organic acids (acetic acid, malic acid, citric acid, gluconic acid and oxalic acid) is the main mechanism of potassium solubilization (Badr, 2006; Sheng and He, 2006; Saiyad et al., 2015). The results of the species and contents of organic acids produced by strain SK1-7 in this study also support this view. Combined with previous studies, it is possible that organic acids secreted by the strain SK1-7 during growth and metabolism will decrease the pH value of potassium solubilization fermentation medium and the pH value of poplar rhizosphere soil. And organic acid-related genes, such as acetic acid kinase *ackA* (Enjalbert et al., 2017) and citric acid synthase *gltA*, which is involved in citric acid synthesis, and malate dehydrogenase *mdh* and phosphoenolpyruvate carboxylase (PEPC) *ppc*, which promote malic acid synthesis, were screened from the whole genome of strain SK1-7 for gene expression verification (Wang P. et al., 2013; Ferraris et al., 2015). Compared with the expression of organic acid synthesis-related genes when a soluble potassium source was used during culture, the expression of these genes when an insoluble potassium source (potassium feldspar) was used was significantly upregulated. This result indicates that strain SK1-7 will secrete more organic acids to participate in potassium hydrolysis under culture with an insoluble potassium source, in which *mdh*, *ppc* and *gltA* participate in the tricarboxylic acid cycle (TCA cycle). It is speculated that strain SK1-7 increases the system energy by enhancing the intracellular TCA cycle to obtain more types and contents of organic acids to dissolve insoluble potassium (Jun et al., 2011).

When microorganisms secrete organic acids to dissolve minerals, they can also secrete extracellular polymeric substances (EPSs), which are mainly composed of proteins and polysaccharides. EPSs can interact with mineral particles to form bacteria-mineral complexes (Wu et al., 2007). In this study, the polysaccharide content secreted by strain SK1-7 under an insoluble potassium source was higher than that under a soluble potassium source. The expression of the *epsB* gene was significantly upregulated with the addition of an insoluble potassium source, and it was upregulated 9.55 times at 10 h. *epsB* is the key gene regulating extracellular polysaccharide synthesis (Elsholz et al., 2014; Liu et al., 2017). When cultured to the later growth of bacteria, it began to secrete a large amount of EPSs, which has a certain adhesion and can be used as an intermediate medium between potassium bacteria and mineral particles to bond them together to form a bacterial-mineral complex. The formation of the bacterial-mineral complex adsorbs the secondary metabolites produced by bacteria, thus changing the microenvironment around minerals (such as pH value), which lead to the release of K^+^. EPSs can also effectively adsorb mineral elements, reduce the ion concentration of these elements, and drive the mineral dissolution equilibrium toward dissolution. The potassium solubilization mechanism of potassium bacteria on potassium-bearing aluminosilicate minerals is based on the formation of bacteria-mineral complexes, which are the result of a variety of factors, including acid hydrolysis, chelation, dissolution and active absorption. These factors promote each other and jointly lead to the gradual precipitation of K^+^ in minerals (Lian et al., 2020).

In previous studies, based on the potassium solubilizing ability of the SK1-7 strain, its potassium solubilizing effect in different mediators was analyzed (Chen et al., 2020). In this study, combined with the previous results, we further explored the potassium solubilizing mechanism of strain SK1-7 and analyzed its potassium solubilizing characteristics, which can provide theoretical support for its development and application as a biological potassium fertilizer. Transcriptome and gene knockout can be used in the future to explain the potassium-solubilizing mechanism of *B. aryabhattai* SK1-7. And the fermentation technology of SK1-7 and the compounds of bacteria and fertilizer need further study.

## Data Availability Statement

The datasets presented in this study can be found in online repositories. The names of the repository/repositories and accession number(s) can be found below: NCBI (accession: PRJNA716807).

## Author Contributions

YC performed the majority of the experiments and data analysis and drafted the link content of the manuscript in the manuscript. JY participated in the planning of research work, interpretation of data, and supervision of manuscript writing. HY and ZS involved in the planning and execution of the research, analysis, and interpretation of the data. All authors read and agreed to the published version of the manuscript.

## Conflict of Interest

The authors declare that the research was conducted in the absence of any commercial or financial relationships that could be construed as a potential conflict of interest.

## Publisher’s Note

All claims expressed in this article are solely those of the authors and do not necessarily represent those of their affiliated organizations, or those of the publisher, the editors and the reviewers. Any product that may be evaluated in this article, or claim that may be made by its manufacturer, is not guaranteed or endorsed by the publisher.

## Footnotes

1
http://crispr.i2bc.paris-saclay.fr/Server/
